# Reactivity against Complementary Proteinase-3 Is Not Increased in Patients with PR3-ANCA-Associated Vasculitis

**DOI:** 10.1371/journal.pone.0017972

**Published:** 2011-03-17

**Authors:** Henko Tadema, Cees G. M. Kallenberg, Coen A. Stegeman, Peter Heeringa

**Affiliations:** 1 Department of Rheumatology and Clinical Immunology, University Medical Center Groningen, University of Groningen, Groningen, The Netherlands; 2 Department of Nephrology, University Medical Center Groningen, University of Groningen, Groningen, The Netherlands; 3 Department of Pathology and Medical Biology, University Medical Center Groningen, University of Groningen, Groningen, The Netherlands; Sheba Medical Center, Israel

## Abstract

The etiology of anti-neutrophil cytoplasmic antibodies (ANCA) associated vasculitides (AAV) is unknown, but the association between infections and autoimmunity has been studied extensively. In 2004, a novel theory was proposed that could link infection and autoimmunity. This ‘theory of autoantigen complementarity’ was based on the serendipitous finding of antibodies against complementary-PR3 (cPR3) in patients with PR3-ANCA-associated vasculitis. cPR3 demonstrated homology to several bacterial proteins, and it was hypothesized that PR3-ANCA develop in response to anti-cPR3 antibodies, as a consequence of the anti-idiotypic network. These data have not been confirmed in other patient cohorts. We investigated the presence of anti-cPR3 antibodies in a Dutch cohort of PR3-ANCA-associated vasculitis patients. Anti-cPR3 reactivity was determined in serum using ELISA. Two separate batches of cPR3 were used to determine reactivity in two separate cohorts of PR3-ANCA-associated vasculitis patients. We found that anti-cPR3-reactivity was not increased in our PR3-ANCA-associated vasculitis patients, in comparison to control groups. Further research will be necessary to prove the concept of autoantigen complementarity in autoimmune diseases.

## Introduction

Anti-neutrophil cytoplasmic antibodies (ANCA) associated vasculitides (AAV) affect small- to medium-sized blood vessels, leading to damage to upper and lower airways, kidneys and other organs. In Wegener's granulomatosis (WG), a prototype AAV, ANCA are mainly directed against proteinase 3 (PR3) [1], [2].

The etiology of WG is unknown, but it has been hypothesized that WG could be triggered by a bacterial or viral infection. Sixty-three percent of patients with WG are chronic nasal carriers of *Staphylococcus aureus* and carriage is associated with an increased risk for relapses [3]–[7]. The development of cross-reactive antibodies as a result of molecular mimicry has been suggested as a mechanism to connect infections and autoimmunity [4], [8]–[10], and recent studies suggest a role for molecular mimicry in ANCA-associated vasculitis. In patients with focal necrotizing glomerulonephritis, Kain *et al.* found autoantibodies against lysosome-associated membrane protein-2 (LAMP-2), which cross-reacted with bacterial FimH, suggesting that anti-LAMP-2 antibodies could be the consequence of a cross-reactive anti-FimH response [11]. Another theory was proposed by Pendergraft *et al.* after they accidentally found anti-idiotypic antibodies in patients with PR3-ANCA-associated vasculitis [12]. Anti-idiotypic antibodies are developed against variable regions of other antibodies and are suggested to play a role in immune regulation and immunological memory [13]–[15]. In 7 out of 34 patients with PR3-ANCA-associated vasculitis, Pendergraft *et al.* found antibodies binding to a protein complementary to the middle part of PR3, and therefore named cPR3m [12]. cPR3m-immunized mice developed both anti-cPR3m antibodies and PR3-ANCA, demonstrating that cPR3m could induce the formation of PR3-ANCA *in vivo*. They hypothesized that a mimic of cPR3m could induce the production of anti-cPR3m antibodies, and subsequently PR3-ANCA via the anti-idiotypic network. Potential cPR3m-homologous structures were found in various pathogens, and it was proposed that a cPR3m-homologous microbial protein could initiate the autoimmune response against PR3 [12].

Although these studies presented promising data that could mechanistically connect infections and PR3-ANCA-associated vasculitis, these findings await confirmation from other patient cohorts. Here we present data on antibody reactivity against cPR3m in patients with ANCA-associated vasculitis.

## Materials and Methods

### Patients

Anti-cPR3m reactivity was determined in two separate cohorts of patients with PR3-ANCA associated vasculitis. In-house produced cPR3m was used to test anti-cPR3m reactivity in serum from 57 ANCA-associated vasculitis patients, and 24 age-and-sex-matched healthy individuals. The study was approved by the Medical Ethics Committee of the University Medical Center Groningen. Written consent was given by the participants for their serum samples to be stored and to be used for research. Patient characteristics are shown in table 1. Diagnoses were based on the Chapel Hill Consensus Conference definitions. A second selection of PR3-ANCA-associated vasculitis patients was tested for anti-cPR3m reactivity, using cPR3m kindly provided by Dr. G. Preston (Chapel Hill, USA). Sera from 37 consecutive PR3-ANCA-positive Wegener's granulomatosis patients (median ANCA titer 1∶160, range 1∶20-1∶640) were analyzed in this assay. cPR3m-reactivity in these patients was compared to that in 21 age-and sex-matched healthy controls.

**Table 1:** Patient characteristics.

ANCA specificity	PR3-ANCA	MPO-ANCA
Total number (diagnosis/follow-up)	34 (25/9)	23 (18/5)
Men/women	19/15	11/12
Age (mean ± sd)	59±15	62±12
Diagnosis		
Wegener's granulomatosis	31	4
Microscopic polyangiitis	3	14
Necrotizing Crescentic Glomerulonephritis		5
BVAS (median/range)		
diagnosis samples	21 (6–33)	17 (9–29)

ANCA: Anti-neutrophil cytoplasmic antibodies.

PR3: Proteinase 3.

MPO: Myeloperoxidase.

BVAS: Birmingham Vasculitis Activity Score.

### Recombinant cPR3m

cPR3m was produced using cPR3m plasmid DNA, kindly provided by Dr. Preston [12]. Purified cPR3m was visualized by Coomassie blue staining after SDS-PAGE. In addition to in-house produced cPR3m, we used cPR3m that was kindly provided by Dr. Preston [12].

### cPR3m Enzyme-Linked Immuno Sorbent Assay (ELISA)

cPR3m ELISAs were performed according to the protocol as described by Pendergraft *et al.* with minor modifications [12]. Briefly, Corning Costar 9018 High Binding ELISA plates were coated with cPR3m (5 µg/ml) in carbonate buffer. Plates were washed with PBS/0.05% Tween-20 and blocked for 1 hour with PBS/1% BSA/0.05% Tween-20 (incubation buffer). Plates were washed and serum samples (diluted 1∶100 in incubation buffer) were incubated 2 h at room temperature. Binding of anti-cPR3m antibodies was detected by alkaline phosphatase labeled anti-human IgG (Sigma). Optical density was measured 60 minutes after adding p-nitrophenyl phosphate substrate at 405 nm.

### Antibodies

Rabbit-anti-cPR3 and chicken-anti-cPR3 antibodies were kindly provided by Dr. Preston, and used as positive controls in cPR3m-ELISAs. Monoclonal anti-HIStag-antibody was obtained from Qiagen.

### Nasal carriage of Staphylococcus aureus

ANCA-associated vasculitis patients who visit our outpatient clinic are routinely tested for nasal carriage of *S. aureus* as described before [6].

### Statistics

Statistical analyses were performed using Graphpad Prism 5.0. The nonparametric Mann-Whitney U test was used to compare anti-cPR3m reactivity between groups. *P* values lower than 0.05 (2-tailed) were considered significant.

## Results

### Characterization of cPR3m

**Figure 1:**
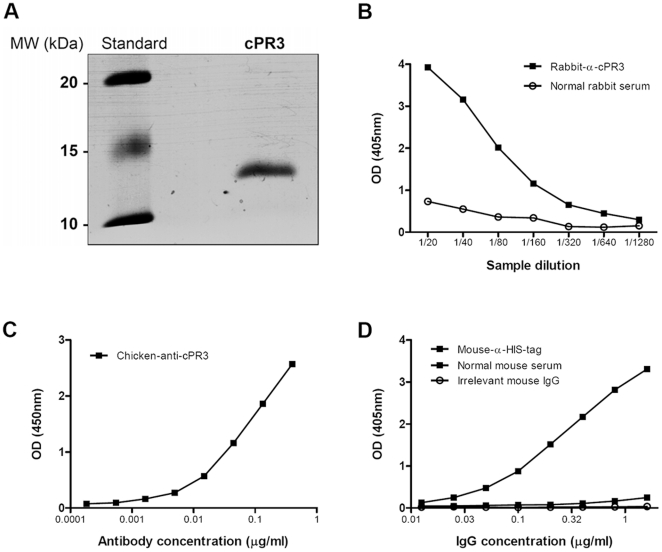
Characterization of in-house produced cPR3m. cPR3m was produced using cPR3 plasmid-DNA provided by Dr. Preston. The protein was purified and visualized by coomassie blue staining after SDS-PAGE. The approximate molecular weight of the protein was 13 kDa. Both Rabbit-α-cPR3 (B) and Chicken-α-cPR3 (C) antibodies bound in a concentration dependant manner to cPR3m in ELISA. D) Binding of mouse-α-HIStag antibody to cPR3m in ELISA.

Purified cPR3m was visualized by Coomassie blue staining after SDS-PAGE, and detected at a molecular weight of approximately 13 kDa (figure 1A). A rabbit-α-cPR3m (figure 1B), a chicken-α-cPR3m, and a mouse α-HIS-tag antibody (figure 1C, 1D) were found to specifically bind purified cPR3m protein in ELISA, indicating proper production and purification of the protein.

### Anti-cPR3m reactivity in AAV patients

**Figure 2:**
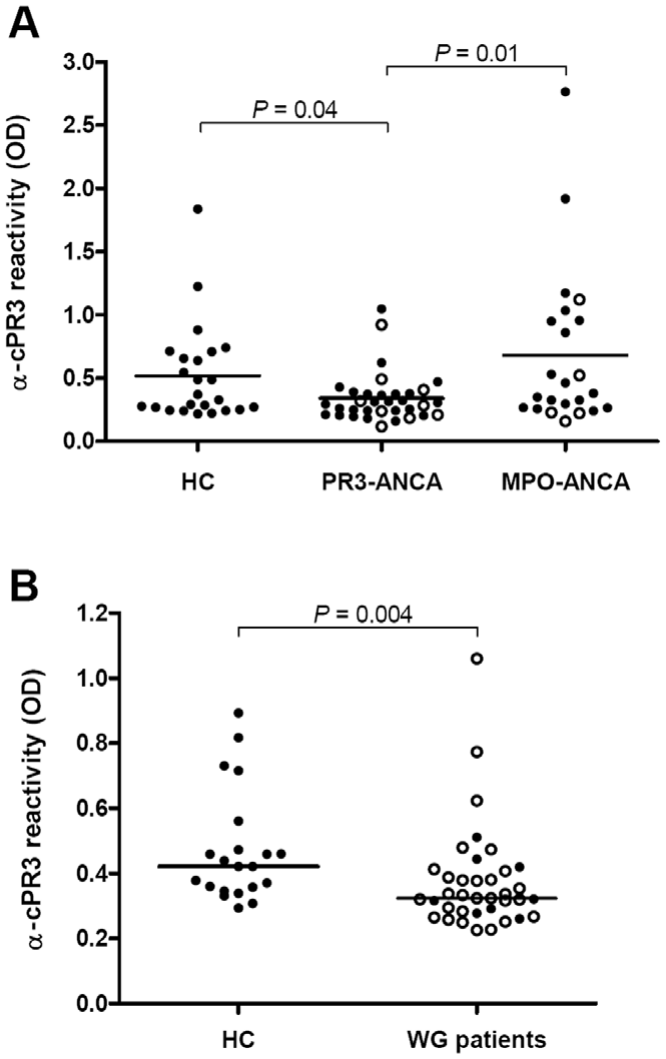
Anti-cPR3m reactivity in serum of AAV patients and healthy controls. A) Anti-cPR3 reactivity in ANCA-associated vasculitis patients and controls, determined by ELISA using in-house produced cPR3m. Anti-cPR3m-reactivity in PR3-ANCA positive patient sera was significantly lower than reactivity in healthy controls (HC) (*P* = 0.04) and MPO-ANCA patients (*P* = 0.01). Anti-cPR3m reactivity in MPO-ANCA positive patients and HC was comparable. In neither PR3-ANCA nor MPO-ANCA positive patients, significant differences were found between samples at the time of disease diagnosis (closed circles) or during follow-up (open circles). B) Anti-cPR3 reactivity in PR3-ANCA positive Wegener's granulomatosis patients and HC, determined by ELISA using cPR3 provided by Dr. Preston. Anti-cPR3m reactivity was decreased in patients, compared to HC (*P* = 0.004). Reactivity in patients at the time of diagnosis (closed circles) did not differ from reactivity in patients in remission.

Anti-cPR3m reactivity in AAV patient serum samples and healthy controls (HC) was determined by ELISA, using in-house produced cPR3m. Anti-cPR3m reactivity was significantly decreased in PR3-ANCA positive patients, compared to both HC (figure 2A, *P* = 0.04) and MPO-ANCA positive patients (figure 2A, *P* = 0.01). Anti-cPR3m reactivity did not differ between patients at diagnosis (closed circles) or during follow-up (open circles) of the disease.

In addition to in-house produced cPR3m, we used cPR3m provided by Dr. Preston to determine anti-cPR3m reactivity in a cohort of 37 consecutive PR3-ANCA positive WG patients. In line with previous results, anti-cPR3m antibody reactivity in patients was significantly lower than reactivity in HC (figure 2B, *P* = 0.004). Anti-cPR3m reactivity did not differ between active patients (closed circles) and patients in remission (open circles).

To study possible idiotypic interactions between anti-cPR3m antibodies and PR3-ANCA, we studied whether sera with high PR3-ANCA titers could inhibit anti-cPR3 reactivity in high-cPR3 reactive sera. However, pre-incubation with neither single PR3-ANCA sera, nor isolated PR3-ANCA IgG samples reduced cPR3m-reactivity in high-cPR3m reactive sera, indicating that PR3-ANCA did not bind anti-cPR3m antibodies in these assays (data not shown).

We investigated a possible relation between nasal carriage of *S. aureus* and the presence of anti-cPR3m antibodies. Anti-cPR3 reactivity in sera from nasal *S. aureus* carriers (median OD 0.37, range 0.12–2.76) did not differ significantly from reactivity in non-carriers (median OD 0.30, range 0.16–1.17).

## Discussion

In 2004, the ‘theory of autoantigen complementarity’ was presented, proposing that anti-idiotypic antibodies could play a role in the development of autoimmune diseases. The theory was based on the observation of anti-cPR3m antibodies in patients with PR3-ANCA-associated vasculitis [12], [16], [17]. So far, this finding has not been confirmed by others. The aim of our study was to investigate the presence of anti-cPR3m antibodies in a different cohort of patients with ANCA-associated vasculitis, in order to confirm data on this new type of antibody.

We successfully produced cPR3m protein in our laboratory. Quality of the cPR3m was tested by ELISA using heterologous anti-cPR3m antibodies. Both rabbit-anti-cPR3m and chicken-anti-cPR3m antibodies reacted strongly with our cPR3m preparation in ELISA.

Having produced and validated cPR3m, reactivity against cPR3m was determined in ANCA-associated vasculitis patients. Anti-cPR3m reactivity was significantly decreased in PR3-ANCA associated vasculitis patients, in comparison to both healthy controls (HC) and MPO-ANCA associated vasculitis patients. The assumption that cPR3m mimics a bacterial protein may explain anti-cPR3m reactivity in control groups. Since the immune system is continuously challenged by pathogens, antibodies against a cPR3m-mimic may have been developed. However, Pendergraft *et al.* found anti-cPR3m reactivity in neither healthy controls nor MPO-ANCA positive patients, supporting the hypothesis that anti-cPR3m antibodies are specifically present in patients with circulating PR3-ANCA [12], [16]. The ‘theory of autoantigen complementarity’ hypothesized that anti-cPR3m antibodies may initiate disease in PR3-ANCA associated vasculitis. Therefore, higher anti-cPR3m levels may be present during the early phase of disease. However, we found comparable anti-cPR3m reactivity in serum samples taken at disease diagnosis and during disease follow-up.

Although the in-house produced cPR3m demonstrated good reactivity with anti-cPR3 antibodies in ELISA, we performed an additional assay, using cPR3m provided by Dr. Preston, to determine anti-cPR3m reactivity in our patient cohort. In this assay we analyzed 37 consecutive Wegener's granulomatosis patients with a positive PR3-ANCA titer. In line with our former finding, this separate group of PR3-ANCA positive vasculitis patients had significantly lower anti-cPR3m reactivity than HC.

The fact that we observed decreased anti-cPR3m reactivity in two separate groups of PR3-ANCA positive patients is intriguing and in contrast with the observations by Pendergraft *et al.* who demonstrated anti-cPR3m antibodies in 7 out of 34 patients with PR3-ANCA associated vasculitis. In the same study they showed that anti-cPR3m and anti-PR3 antibodies form an idiotypic pair [12]. Circulating anti-idiotypic antibodies have been observed in AAV patients and it has been shown that antibody reactivity may be masked by idiotypic antibodies [18]–[21]. Therefore, formation of PR3-ANCA – anti-cPR3 complexes may have masked anti-cPR3m reactivity in our assays. On the other hand, Pendergraft *et al.* selected PR3-ANCA positive patients as well, and did find anti-cPR3 antibodies, although PR3-ANCA titers in these patients were not described. We studied possible idiotypic complex formation *in vitro*, but were not able to demonstrate this.

Proteins from several pathogens were suggested to mimic cPR3m, amongst which two proteins from *Staphylococcus aureus* [12]. Development of antibodies against proteins from *S. aureus*, in particular by nasal carriers, has been shown [22], [23]. Therefore, we investigated a possible relationship between nasal carriage of *S. aureus* and anti-cPR3m reactivity in AAV patients. Reactivity against cPR3m did not differ significantly between nasal carriers and non-carriers of the bacterium.

In summary, we conclude that, analyzed by the methods as described here, reactivity against cPR3m was not increased in our PR3-ANCA-associated vasculitis patients. In fact, we found significantly decreased anti-cPR3m reactivity in PR3-ANCA-associated vasculitis patients, compared to both HC and MPO-ANCA patients. This intriguing result may be explained by the hallmark characteristic of anti-idiotypic antibodies that they recognize each other, thereby masking anti-cPR3 reactivity, but we were not able to prove this. Further research is necessary to increase the understanding on anti-cPR3 antibodies in patients with PR3-ANCA associated vasculitis and to conclude about the possible role of anti-idiotypic antibodies in the development of ANCA-associated vasculitis.
